# Complex Analysis of Vanillin and Syringic Acid as Natural Antimicrobial Agents against *Staphylococcus epidermidis* Biofilms

**DOI:** 10.3390/ijms23031816

**Published:** 2022-02-05

**Authors:** Andrej Minich, Zdenko Levarski, Mária Mikulášová, Marek Straka, Adriána Liptáková, Stanislav Stuchlík

**Affiliations:** 1 Faculty of Natural Sciences, Comenius University in Bratislava, 84104 Bratislava, Slovakia; minich5@uniba.sk (A.M.); zdenko.levarski@uniba.sk (Z.L.); mutiska@gmail.com (M.M.); 2 Science Park, Comenius University in Bratislava, 84104 Bratislava, Slovakia; 3 Faculty of Medicine, Comenius University in Bratislava, 81372 Bratislava, Slovakia; marek.straka@fmed.uniba.sk (M.S.); adriana.liptakova@fmed.uniba.sk (A.L.)

**Keywords:** *Staphylococcus epidermidis*, biofilm, quorum sensing, vanillin, syringic acid

## Abstract

The presence of *Staphylococcus epidermidis* biofilms on medical devices is a major cause of nosocomial diseases and infections. Extensive research is directed at inhibiting the formation and maturation of such biofilms. Natural plant-derived phenolic compounds have promising antimicrobial effects against drug-resistant bacteria. The anti-biofilm activity of two selected phenolic compounds (vanillin and syringic acid) was tested against three biofilm-forming methicillin-resistant *S. epidermidis* strains with different genotypes. Resazurin assay combining crystal violet staining and confocal microscopy was used for biofilm and extracellular polymer substance (EPS) inhibition tests. Effects on EPS compounds such as proteins, extracellular DNA, and polysaccharides were also examined. Combined with quantitative real-time PCR of selected *agr* quorum-sensing systems and biofilm genetic determinants, our complex analysis of vanillin and syringic acid showed similar biofilm and EPS inhibition effects on *S. epidermidis* strains, reducing biofilm formation up to 80% and EPS up to 55%, depending on the genotype of the tested strain. Natural antimicrobial agents are thus potentially useful inhibitors of biofilms.

## 1. Introduction

*Staphylococcus epidermidis* is an opportunistic pathogen of human microflora. In contrast to *Staphylococcus aureus*, which expresses a broad spectrum of virulence factors, the main strategy of *S. epidermidis* is biofilm formation leading to the spread of chronic or acute infections [1,2]. Biofilm is one of the survival strategies of bacterial communities [3] and is defined as a multicellular community of bacteria coated with an extracellular polymer matrix (EPS) composed of polysaccharides, proteins, extracellular DNA (eDNA), lipids, and other components [4]. EPS is one of the most important factors in the survival of the biofilm structure since it protects biofilms from drying out, from UV radiation, and from other extracellular factors [5]. The composition of the EPS changes as influenced by the external environment conditions defines the type of biofilm formed by Staphylococci [6].

Among the regulatory mechanisms that ensure the dynamic adaptation of the physiology of the staphylococcal biofilms to the environment, quorum sensing (QS) is one of the most studied and probably the most important mechanisms for controlling pathogenesis and virulence [7]. QS as a cellular communication system used by bacterial pathogens coordinates biofilm formation and the production of virulence factors [8,9]. Expression of QS genes is energy-intensive for the cell and is induced only when the cells reach high density [10]. In *S. epidermidis*, the *agr* (accessory gene regulator) system is considered a prototype of the QS mechanism. The fundamental features of the *agr* QS system are highly conserved among *Staphylococcus species*, especially in *S. epidermidis* [11]. The *agr* locus of the QS mechanism is approximately 3.5 kb in size and consists of two transcription units, namely RNAII and RNAIII, driven by the promoters P2 and P3 [12]. The P3 promoter controls the production of the 510-nucleotide-long RNAIII intracellular effector molecule of the *agr* QS system [13] and is responsible for the regulation of the system itself [14] and of many virulence factors and biofilm proteases such as EcpA, EspA, and SepA [15]. It has also been characterized as a mediator RNA (mRNA) for the delta-toxin (delta-hemolysin) *hld* gene, which enhances *S. epidermidis* virulence [16]. The P2 promoter controls the transcription part of the *agr* locus consisting of four genes, namely the *agrB, agrD, agrC,* and *agrA* response regulator genes controlling the *agr* QS system [17,18]. The agrA response regulator up-regulates the transcription of phenol-soluble modulin (PSM) genes via the *psmα* and *psmβ* operons by binding to appropriate promoter sequences [19,20]. PSMs play an important role in cytotoxin function and in the stimulation of inflammatory responses, such as chemotaxis or the induction of cytokine expression, and are involved in biofilm formation [21]. PSMβ supports biofilm structure, biofilm dispersion, and the in vivo spreading of biofilm-induced infections. However, no direct information has as yet been found with regard to the function of other phenol-soluble modulins, mainly because of the complexity of their analysis [22].

In recent years, increased interest has been shown in plant phenolic compounds because of their potential as suitable anti-microbials and anti-biofilm agents. The main reason for this interest is that the use of anti-microbials from natural sources does not lead to the development of bacterial resistance [23,24] since these compounds are used to control bacterial infections targeting virulence factors or biofilm development through lowering QS cell to cell communication rather than the viability of bacterial cells [24]. Phenolic compounds are known for their antioxidant effects and as secondary plant metabolites and can be divided into several classes, including phenolic acids, flavonoids, and tannins [25]. Although the primary structure is the same in almost all phenolic compounds, their biological properties are mainly influenced by the position and number of hydroxyl groups in the phenolic ring [26,27]. Phenolic compounds have been shown to be effective in inhibiting biofilm formation and cell growth in pathogenic bacteria [28]. Some phenolic substances, such as vanillin, syringic acid, eugenol, carvacrol, and the flavonoid tiliroid, have modulatory effects on antibiotic resistance in *S. aureus* and *S. epidermidis* by inhibiting the functioning of efflux pumps, which are one of the important factors in biofilm maturation [29], which was a main criterium in the selection of potential antimicrobial phenolic compounds in this work.

In this study, vanillin and syringic acid were selected from several phenolic compounds isolated from oak bark lignin waste for the determination of their anti-biofilm activity on three *S. epidermidis* clinical strains with different genotypes. Confocal microscopy in combination with microbiological methods was used to analyze changes in EPS compositions and was supported by real-time qPCR.

## 2. Results

### 2.1. Selection of Phenolic Compounds

**Figure 1:**
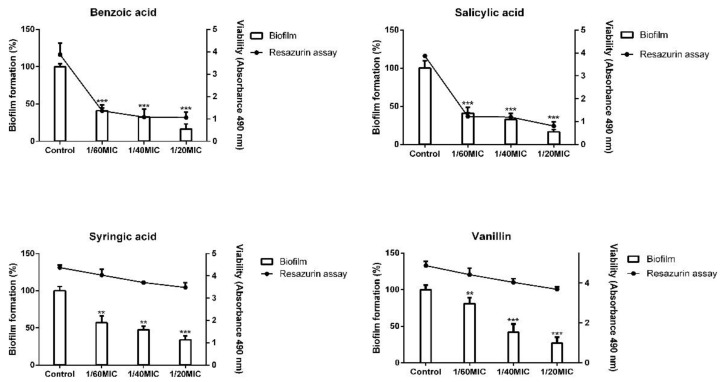
Analysis of non-antibacterial effect of 4 selected phenolic compounds. Based on MICs, a combination of resazurin assay for viability determination and biofilm formation assay with crystal violet was used on the representative strain *S. epidermidis* RP62A (one-way ANOVA (**: *p* < 0.01, ***: *p* < 0.001)).

In our preliminary screening, vanillin, syringic acid, salicylic acid, and benzoic acid showed dose-dependent inhibition in three subinhibitory concentrations (results not shown). Since the minimal inhibition concentrations (MICs) for all four analyzed phenolic compounds were determined at different concentrations, subinhibitory concentrations are indicated in this work as 1/60, 1/40, and 1/20 MIC. To determine the non-antibacterial effect of the selected compounds, a resazurin assay in combination with biofilm formation analysis was carried out on the representative strain *S. epidermidis* RP62a (Figure 1). Staining with resazurin dye had no effect on further staining with crystal violet, allowing us to perform viability and biofilm formation assays within the same bacterial culture.

The results showed that, for vanillin and syringic acid, the employed subinhibitory concentrations did not substantially influence cell viability. On the contrary, salicylic acid and benzoic acid markedly lowered viability with a concomitant effect on biofilm formation at all three used concentrations compared with the control.

**Figure 2:**
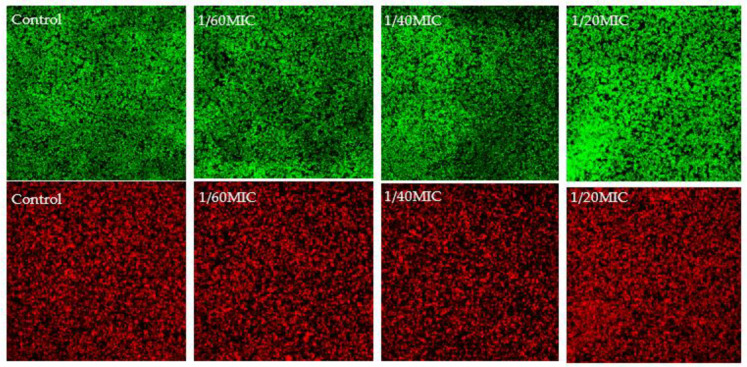
Analysis of non-antibacterial effect of vanillin by using CLSM. Staining of biofilms of *S. epidermidis* RP62a after 24 h was influenced by vanillin at selected subinhibitory concentrations. As a control, a bacterial biofilm with no vanillin was used.

**Figure 3:**
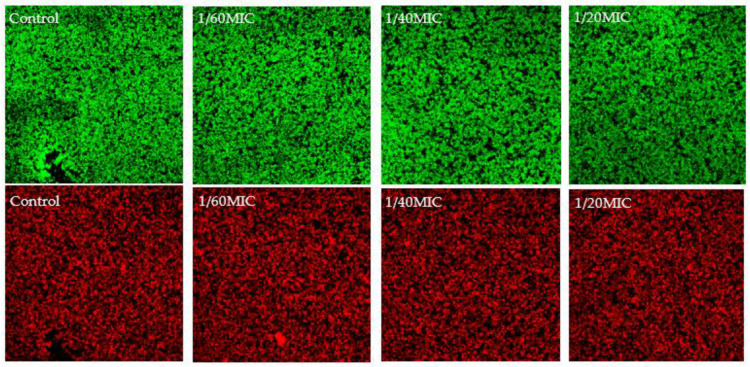
Analysis of non-antibacterial effect of syringic acid using CLSM. Staining of biofilms of *S. epidermidis* RP62a after 24 h was influenced by syringic acid vanillin at selected subinhibitory concentrations. As a control, a bacterial biofilm with no syringic acid was used.

To confirm the viability shown in the resazurin assay for vanillin and syringic acid, confocal laser scanning microscopy (CLSM) was performed using DioC6 (green color) to stain viable cells and propidium iodide (red color) to stain dead cells (Figure 2 and Figure 3). Our results showed a correlation between the two methods.

The ratio between dead and live cells did not increase for all used subinhibitory concentrations of both phenolic compounds. These results led us to select vanillin and syringic acid for further analysis as potential anti-biofilm and anti-QS agents.

### 2.2. Biofilm Formation Inhibition Using Phenolic Compounds

**Figure 4:**
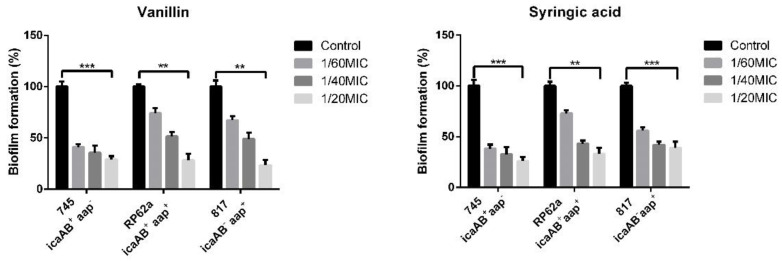
Influence of vanillin and syringic acid on biofilm formation in microtiter well plates; crystal violet staining. Three *S. epidermidis* strains with different biofilm genetic determinants (745, RP62a, and 817) were used to analyze potential anti-biofilm effects of vanillin and syringic acid at subinhibitory concentrations. Biofilm mass formation was inhibited in a dose-dependent manner (one-way ANOVA (**: *p* < 0.01, ***: *p* < 0.001)).

The potential anti-biofilm effect of vanillin and syringic acid was verified by the crystal violet staining protocol for staphylococci. We examined selected *S. epidermidis* strains with different biofilm determinant genotypes (*icaAB/aap*), namely 745, RP62a, and 817. Both vanillin and syringic acid showed a dose-dependent inhibition for all three *S. epidermidis* strains (see Figure 4).

Vanillin allowed only 29% of 745 clinical strain biofilm formation at the highest used concentration 1/20MIC compared with the control. Similar results were found with vanillin for strains RP62A and 817, in which inhibition ranged from 73% to 78%. Syringic acid inhibited biofilm formation and had the same effect as vanillin, allowing biofilm formation by the 745 clinical strain only up to 25% at 1/20MIC. In the presence of syringic acid at the highest used subinhibitory concentration biofilm, strain RP62a formed a biofilm up to 30% and strain 817 up to 40% compared with the control.

### 2.3. EPS Formation Inhibition Using Phenolic Compounds

**Figure 5:**
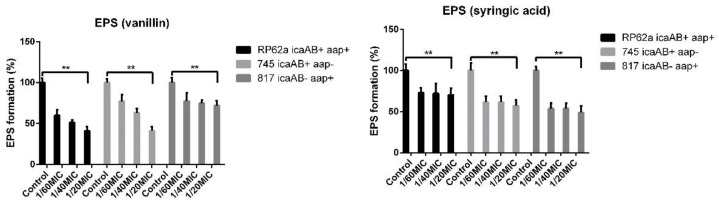
WGA assay testing of vanillin and syringic acid on EPS formation under static biofilm conditions in microtiter 96-well plates. Results show an inhibition trend for all used concentrations independently of genotype of tested strain (one-way ANOVA (**: *p* < 0.01)).

WGA assay was used to determine the inhibition effect on EPS of matured biofilm in 96-well plates (Figure 5). As shown, vanillin and syringic acid inhibited EPS polysaccharide production in all tested strains. For RP62A EPS, vanillin markedly inhibited polysaccharide production and reduced EPS by about 60% at the 1/20MIC concentration. Syringic acid reduced EPS by about 40%. In the case of the 745 clinical strain, vanillin provided 60% inhibition, and syringic acid caused about 30% inhibition, The EPS of the 817 clinical strain was inhibited to a maximum of 20% by vanillin or 55% by syringic acid at the 1/20MIC concentration.

### 2.4. Complex Analysis of EPS Components

**Figure 6:**
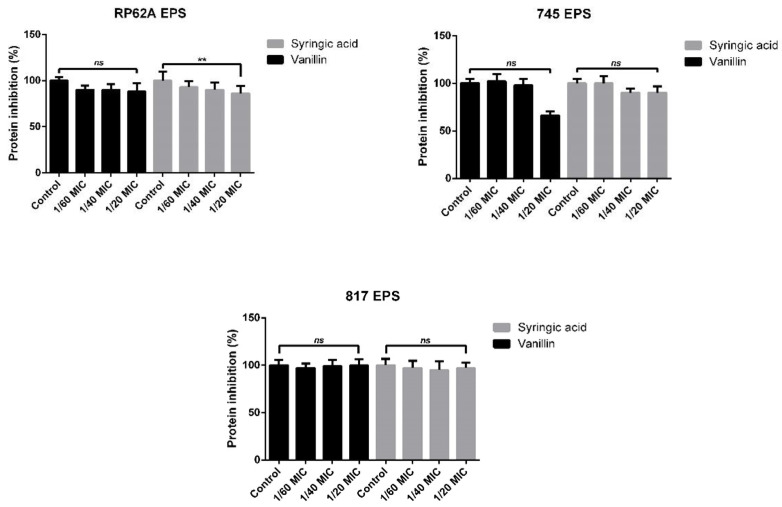
Bradford protein assay for determination of protein concentration in isolated EPS fractions of all tested *S. epidermidis* strains. Protein quantification showed non-significant protein inhibition in EPS fraction when using vanillin and syringic acid at almost all subinhibitory concentrations. As a standard, bovine serine albumin (BSA) was used to determine the concentration of proteins in the samples (one-way ANOVA (** = *p* < 0.01)).

Next, the formed EPS in the presence of vanillin and syringic acid was isolated, and the protein and sugar content were analyzed. The Bradford protein assay was used to determine the protein content for each strain (Figure 6). The protein concentration in the control grown in the absence of either of the phenolic compounds was 200 μg/mL for RP62A and 150–160 μg/mL for the 745 and 817 clinical strains.

None of the used subinhibitory concentrations of vanillin and syringic acid affected protein content significantly (Figure 6). RP62A EPS protein production was inhibited mostly by around 10% for both analyzed phenolic compounds, and the 745 EPS protein mass was reduced by 40%. However, statistical analysis revealed that the change was non-significant. The Bradford assay did not show an inhibition trend for the 817 strain.

**Figure 7:**
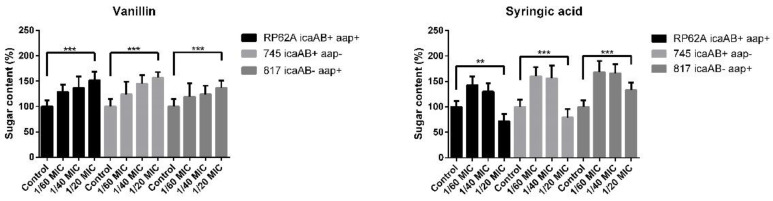
DuBois method for sugar total sugar content in EPS fractions of tested *S. epidermidis* strains. Quantification of sugars showed a correlation between the genotype of the tested strains and the sugars content in the EPS. Strain 745 contained the highest mass of sugars in the EPS. The addition of vanillin enhanced the sugar content in the EPS, whereas syringic acid lowered the sugar mass only at 1/20 MIC. As a control, glucose was used to determine the concentration of sugars in the samples (one-way ANOVA (**: *p* < 0.01, ***: *p* < 0.001)).

The calorimetric DuBois method was used to measure the sugar content in the isolated EPS of the respective biofilms. The total sugar content in the EPS for clinical strain 745 was 1800 μg/mL and, for RP62A, was 1600 μg/mL. The lowest content was determined for clinical strain 817, which had a concentration of sugars of about 600 μg/mL. The data showed that vanillin increased the sugar content in the EPS of all three tested strains independently of genotype (Figure 7).

*S. epidermidis* RP62A EPS in the presence of the highest vanillin concentration contained about 62% more sugars than the control (Figure 7). Similar results were obtained for both the clinical strains 745 and 817, in which the increase in sugars in the isolated EPS ranged from 50% to 70%. Syringic acid significantly enhanced the sugar proportion at the 1/60 MIC and 1/40 MIC concentrations, the highest increase being observed for clinical strain 817, in which the percentage of sugars was 70% higher than that in control. Inhibition of sugar production was determined in RP62A and 745 EPS but was only 20% lower compared with the control in the absence of syringic acid.

**Figure 8:**
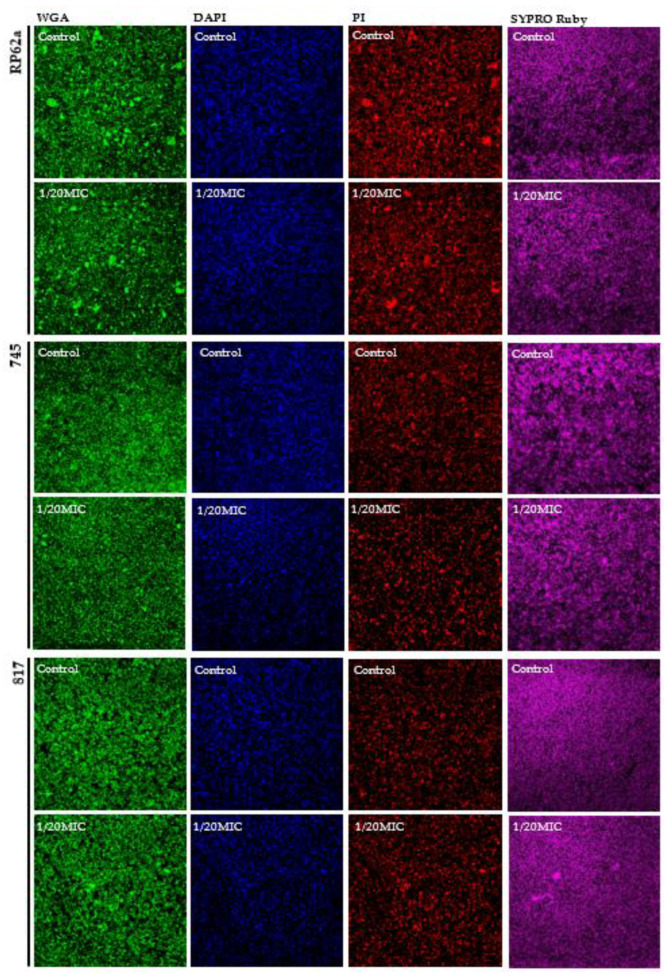
CLSM analysis of the inhibition of EPS biofilm components by vanillin in all three tested *S. epidermidis* strains. PIA(PNAG) was stained with WGA, proteins were stained with SYPRO Ruby, and eDNA was stained with PI. To analyze the inhibition of eDNA, DAPI was used to visualize the contrast between eDNA and the intracellular non-selective PI staining. The potential inhibition of EPS components was determined at the highest used subinhibitory concentration, 1/20MIC, compared with the control.

**Figure 9:**
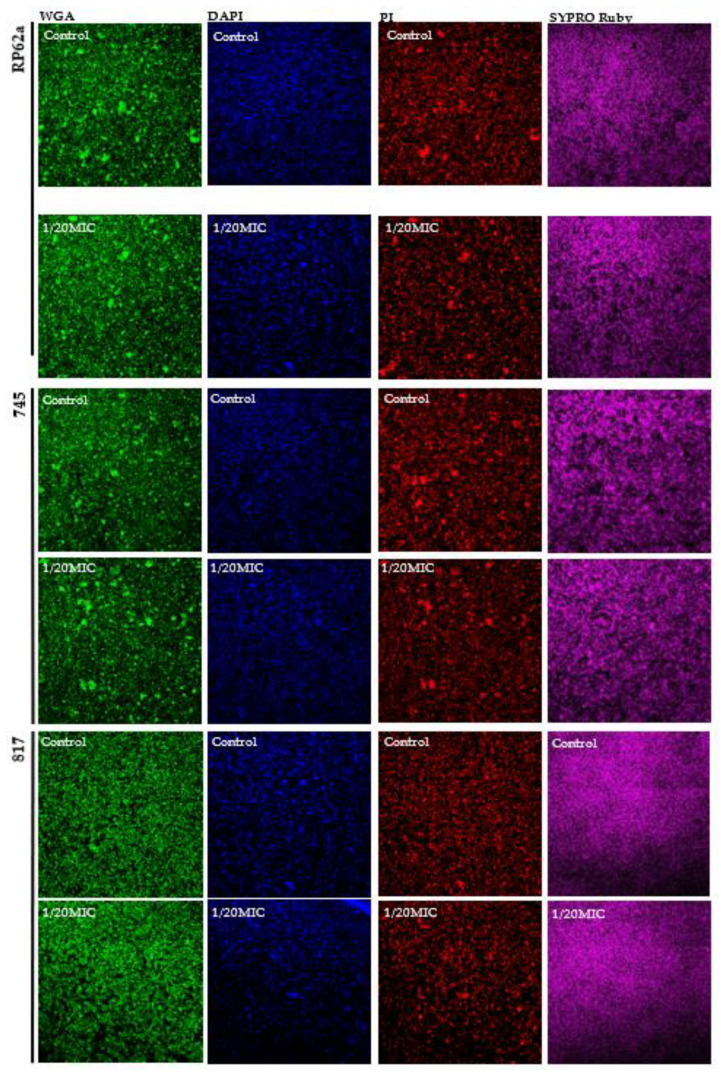
CLSM analysis of inhibition of EPS components of biofilm by syringic acid in all three tested *S. epidermidis* strains. PIA(PNAG) was stained with WGA, proteins were stained with SYPRO Ruby, and eDNA was stained with PI. To analyze the inhibition of eDNA, DAPI was used to visualize the contrast between eDNA and the intracellular non-selective PI staining. The potential inhibition of EPS components was determined at the highest used subinhibitory concentration, 1/20MIC, compared with the control.

Since phenolic compounds can react with the reagents of the DuBois method, and since our main focus was on the detection of poly-N-acetyl-β-(1-6)-glucosamine (PIA/PNAG), changes in the PIA/PNAG content were determined by CLSM. Other components such as eDNA and protein content were analyzed by microscopy. The presence of vanillin and syringic acid showed inhibitory effects at 1/20MIC (Figure 8 and Figure 9). The amount of PIA/PNAG in EPS was significantly reduced for the 745 clinical strain, i.e., up to 27%. Similar inhibition was observed for the RP62a strain and also for 817, in which inhibition was about 15%.

The protein content was not influenced by vanillin or syringic acid in all three analyzed strains; these results correlated with the Bradford protein assay (Figure 6). CLSM showed a similar pattern for the protein distribution in the EPS for the RP62a and 745 strains compared with 817. Whether they were similar because of the genotype (*icaAB+*) is the subject of further research.

### 2.5. Real-Time qPCR of Biofilm Determinants

#### 2.5.1. RNAIII-Dependent Genes

**Figure 10:**
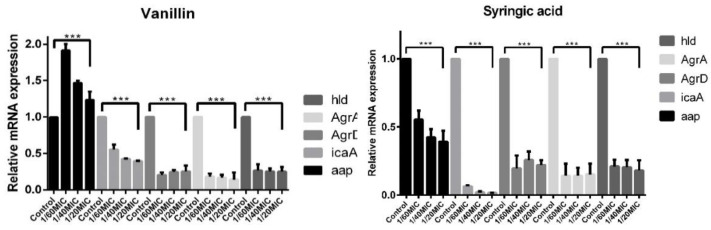
Relative mRNA expression fold change of overall biofilm determinants by using real-time qPCR. With each of the phenolic compounds, for all of the genes, except for the *aap* gene, the relative mRNA expression was lowered compared with the control. The fold change for the analyzed genes ranged from two-fold to three-fold. As a control, a housekeeping gene for 16S rRNA was used (one-way ANOVA (***: *p* < 0.001)).

The relative mRNA expression of biofilm genetic determinants (*icaA, aap*), two representative genes of *agr* QS system (*agrD, agrA*), and the *hld* toxin gene were calculated for both phenolic compounds. With vanillin, all three used concentrations lowered the expression of the four genes (shown in Figure 10). Our results revealed a dose-independent mRNA fold change by two-thirds in comparison with the control for *aap*, *icaA*, *agrD*, and *agrA*. Only *aap* gene expression was two-fold higher. Syringic acid lowered the mRNA expression for all five analyzed genes with the same dose-independent trend. The determined change ranged from almost no expression for the *agrA* gene at the 1/20MIC concentration to half the expression of the *aap* gene compared with the control.

#### 2.5.2. RNAIII-Independent Genes

**Figure 11:**
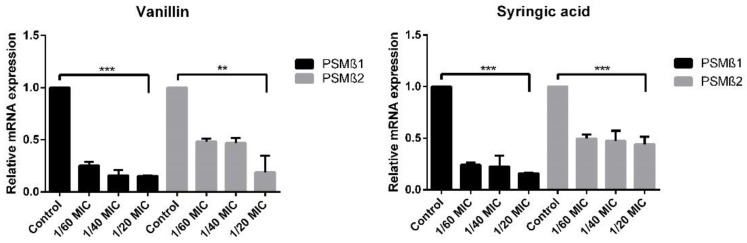
Relative mRNA expression fold change of RNAIII-independent genes PSMβ1 and PSMβ2 by using real-time qPCR. For both the genes, the relative mRNA expression was lowered compared with that of the control by vanillin and syringic acid. As a control, a housekeeping gene for 16S rRNA was used.(**: *p* < 0.01, ***: *p* < 0.001).

The relative mRNA expression of PSMβ1 was lowered by 80% by each of the phenolic compounds at 1/20MIC. The relative mRNA expression of PSMβ2 was lower by 75% at the 1/20MIC concentration of vanillin, and for syringic acid, the expression was two-fold lower compared with the control, resulting in a 50% inhibition of expression (shown in Figure 11).

## 3. Discussion

With the rapid onset of the post-antibiotic era, the identification of new means of fighting against pathogenic bacteria infections represents an essential step toward supporting host defense mechanisms [30,31]. During the last decade, interest in biofilm inhibition by natural compounds has increased because many studies have shown that plant-derived compounds are promising candidates as antimicrobial agents against biofilms and the related quorum-sensing mechanism [32,33]. The aim of anti-biofilm strategies is to intervene in intercellular communication mechanisms such as quorum sensing, via which bacteria regulate gene expression to increase their fitness [30].

In the present study, a complex analysis of vanillin and syringic acid focused on the inhibition of EPS compounds was performed on three *S. epidermidis* strains having different genotypes. Preliminary findings led us to select four phenolic compounds from oak tree bark for closer analysis, namely benzoic acid, vanillin, syringic acid, and salicylic acid, based on their minimal inhibition concentration (MIC). To determine that these phenolic compounds did not act as anti-bacterial agents, a resazurin viability assay was performed on the representative strain *S. epidermidis* RP62a (Figure 1). Unlike the tetrazolium reduction assay involving the use of MTT or XTT [34], the resazurin viability assay allows the test to be combined with other assays such as crystal violet staining [35,36] and was therefore chosen to obtain accurate results. The use of three determined subinhibitory concentrations of each phenolic compound in biofilm culture showed that vanillin and syringic acid did not affect the viability of cells in biofilm structures but was correlated with a lowering of biofilm formation. On the contrary, in the case of benzoic acid and salicylic acid, viability was drastically lowered in all of the three used concentrations (Figure 1).

To support the resazurin viability assay, live/dead staining with DioC6 and propidium iodide (PI) was used to study the effects of vanillin and syringic acid (Figure 2 and Figure 3). For each of these phenolic compounds at all three subinhibitory concentrations, the live/dead ratio did not change and was correlated with the resazurin assay. Since PI stains in a non-selective manner and can label another layer of dead cells because of the double staining of eDNA outside intact membranes and eDNA of biofilm EPS [37], live/dead staining was used only to support the analysis of a potential anti-bacterial effect by the resazurin assay. As our key criterium was that the natural compound did not interfere with any metabolic pathway or affect the viability and had no bactericidal effect on a wider scale of the concentrations used, these findings led us to select only vanillin and syringic acid for further anti-biofilm and anti-QS analysis.

The anti-biofilm and EPS inhibition effects were analyzed using crystal violet staining of three *S. epidermidis* strains with different biofilm genotypes. For all three strains, vanillin inhibited biofilm formation at all used subinhibitory concentrations. The 1/20MIC concentration reduced 745 strain biofilm formation up to 71%. Similar results were observed for strains 817 and RP62a, in which the inhibition ranged around 75%. Syringic acid had the same effect as vanillin, and biofilm mass was reduced by 50%–70%, depending on the strain genotype. For the *S. epidermidis* strain 745, syringic acid had a similar effect at all three concentrations. Similar results for vanillin were observed in *Hafnia alvei* [38], *Pseudomonas aeruginosa* [39], and *Candida albicans* [40], in which biofilm formation was inhibited up to 80%. Syringic acid anti-biofilm and anti-QS potential have not previously been analyzed.

To quantify the influence of vanillin and syringic acid on EPS in biofilms in 96-well microtiter plates, we performed the WGA assay [41]. The limiting factor for the determination of EPS in 96-well microtiter plates using the WGA assay according to the protocol [41] is a minimum number of bacteria 10^7^ CFU/mL. We confirmed that a sufficient concentration of cells was present for 24 h in the *S. epidermidis* biofilm of all tested strains. Each of the phenolic compounds inhibited EPS in all three *S. epidermidis* strains. In the case of the RP62a strain, the EPS was reduced by 60% at the 1/20MIC concentration. The same effect was achieved by vanillin on the 745 strain EPS. The lowest effect of vanillin was determined in strain 817 at all three concentrations. Syringic acid produced a 30% to 60% reduction in EPS, depending on the genotype of the *S. epidermidis* strain. Next, the EPS from the produced biofilms in all three strains treated with vanillin and syringic acid was isolated using 1.5 M NaCl [41], and the protein content and sugar content were determined using the Bradford assay and the DuBois method [42], respectively. Results from the Bradford assay showed that, in the presence of each of the phenolic compounds at all used subinhibitory concentrations, the protein content did not change or did not change significantly. The control RP62a protein content was approximately 200 μg/mL, which was the highest protein concentration in all three strains. At the highest used concentration of 1/20MIC, protein inhibition was only 10%. For strains 817 and 745, the total protein content was determined to be approximately 150–160 μg/mL, with the highest inhibition being found in the 745 strain at around 40%.

We also analyzed the total amount of sugars in the EPS in the presence of vanillin and syringic acid. The total sugar content in the EPS for clinical strain 745 was 1800 μg/mL, for RP62A 1600 μg/mL, and for 817 strain about 600 μg/mL. The presence of vanillin-enhanced sugar content was seen at all used concentrations. Our results showed that the use of the 1/20MIC concentration of vanillin-enhanced sugars in EPS from 50% to 70%, depending on the strain genotype. Syringic acid at 1/60MIC and 1/40MIC significantly influenced sugar mass, with the highest increase of about 70% being observed for strain 817 compared with the control. In general, phenolic compounds with multiple hydroxyl groups exhibit a high redox potential. Moreover, all the phenolic compounds are characterized by a high hydroxyl radical reactivity [43] that can interfere with other pathways in bacteria and that can induce the production of sugars, although more studies are needed with regard to this reactivity.

In general, phenolic compounds act as QS signal inhibitors and can influence the expression of many genes and the composition of EPS. The effect of vanillin and syringic acid on EPS components at the highest used concentration of 1/20MIC compared with the control was analyzed by CLSM (Figure 8 and Figure 9). Proteins were stained with a SYPRO Ruby biofilm matrix stain, poly-N-acetyl-β-(1-6)-glucosamines (PIA/PNAG) were stained with WGA, and eDNA was stained with propidium iodide and DAPI. To support CLSM, the mRNA expression of biofilm genetic determinants was analyzed by real-time qPCR (Figure 10).

Our results showed a substantial difference in the PNAG amount at the 1/20MIC concentration of each of the phenolic compounds compared with the control. This change supported the relative mRNA expression of the *icaA* gene, which was lowered three-fold at 1/20MIC in the case of vanillin. Syringic acid also inhibited the production of PNAG, and almost no expression of the *icaA* gene was seen at the highest concentration. These results differed from those obtained by the DuBois method in which the sugar content was enhanced. However, they agree with the assertion that phenolic compounds present in water have high tendencies of interacting or reacting with other components of the aquatic environment and can influence the production pathways of other components [44], in this case, saccharide production. Since phenolic compounds interfere with proteins and affect their solubility and activity [45,46], this phenomenon might explain (1) the unchanged protein production in all three strains (Figure 8 and Figure 9) seen after using the Bradford protein assay and (2) the different distribution of protein in the EPS of the 745 and RP62a strains in which the proteins seem to be more aggregated (Figure 8 and Figure 9). No change in the protein mass was determined, although *aap* gene relative mRNA expression was influenced by both phenolic compounds, leading to the assumption that other genes were upregulated; however, more analyses are needed. In the presence of vanillin, the expression of the *aap* gene was two-fold higher, and on the contrary, syringic acid lowered the expression by two-fold (Figure 10). In order to analyze eDNA changes in EPS, we used DAPI and PI. DAPI was employed as a counterstain, since PI stains all nucleic acid from dead cells, even in EPS [47], and can create a high fluorescence background [37]. Vanillin and syringic acid showed similar effects, and an inhibition in the amount of eDNA was observed for all three stains, regardless of genotype, on being stained with PI. A positive influence of each of the phenolic compounds on eDNA in the EPS might lead to the easier eradication of biofilms. Similar results have been observed for *S. aureus* [48,49].

The main potential of phenolic compounds is that they have the ability to mimic QS signals [50,51]. The relative mRNA expression of the selected *agr* QS mechanism was analyzed in the presence of vanillin and syringic acid in the representative strain *S. epidermidis* RP62a (Figure 10). Both genes under the P2 promoter, namely *agrA* and *agrD*, were downregulated five-fold compared with the control at all of the three used subinhibitory concentrations. The same result was obtained for the *hld* gene controlled by the P3 promoter. A decrease in signal pathway activation might lead to the formation of fewer rigid biofilms (Figure 4) and might have a high impact on their EPS composition (Figure 5, Figure 8 and Figure 9). The QS inhibition effect of phenolic compounds has also been observed for foodborne bacteria such as *Aeromonas hydrophila* and *Chromobacterium violaceum* by using curcumin and resveratrol [52] or for *P. aeruginosa* in the presence of methyl gallate [53].

The effect of vanillin and syringic acid on the expression of RNAIII-independent genes was also analyzed, as represented by *psmβ1* and *psmβ2* genes. The expression of both genes was markedly lowered five-fold by using vanillin and syringic acid in the highest used concentration (1/20MIC). Only PSMβ2 mRNA expression in the presence of syringic acid was lower by 50%. Interestingly, for all genes, RNAIII-dependent or -independent relative mRNA expression changed in a similar manner regardless of the concentration used.

All the data taken together indicate that vanillin and syringic acid have the potential to act as antimicrobial agents against *S. epidermidis*, independent of the biofilm determinant genotype of the tested strain and that both phenolic compounds have anti-QS and anti-biofilm activity in vitro.

## 4. Materials and Methods

### 4.1. Bacterial Strains and Culture Conditions

In this study, we examined biofilm-producing strain *S. epidermidis RP62A* (ATCC 35984) and two clinical isolates, namely 745 and 817. Strains were maintained in tryptic soy broth (TSB) agar and medium. For assays, a TSB medium supplemented with 1% glucose was used to induce biofilm formation.

### 4.2. Phenolic Compounds

The analyzed phenolic compounds were isolated from oak bark lignin waste and were provided by the Slovak Technical University in Bratislava. Compounds were stored at room temperature and diluted at subinhibitory concentrations in 99.9% dimethylsulfoxide (DMSO). The range of used subinhibitory concentrations in this work was prepared from stock solution at concentration 250 mM/mL for each phenolic compound calculated based on molar weight. Stocks were stored at 4 °C when used for assays.

### 4.3. Assessment of Metabolic Activity of Biofilm by Resazurin

The resazurin metabolic experiments were performed in 96-well microtiter plates. The bacterial culture, together with the analyzed antimicrobial phenolic agents at subinhibitory concentrations, was incubated at 37 °C for 24 h under static conditions. After incubation, the bacterial biofilm was washed with PBS (pH 7.2) to remove planktonic cells and incubated with 200 μL resazurin (4 μg/mL) for 45 min in the dark. Following incubation, a multimode microplate reader Varioskan Flash (Thermo Fisher Scientific, Waltham, MA, USA), was used to measure the relative fluorescence units (RFU) (λEx 530 nm and λEm 590 nm). In combination with biofilm formation analysis, resazurin was removed after the first measurement, and the protocol for crystal violet staining [53] was then followed.

### 4.4. Assessment of Biofilm Formation by Crystal Violet Staining

Biofilm formation and inhibition were analyzed by crystal violet staining for staphylococci [54]. Overnight cultures of the tested strain were diluted with TSB supplemented with 1% glucose at a 1:100 ratio, and a 200 μL bacterial culture was poured into the well. The optical density (OD) was measured at 570 nm by using a microtiter plate reader (Varioskan Flash, Thermo Fisher Scientific, Waltham, MA, USA).

### 4.5. EPS Inhibition Analysis by WGA Assay

Bacterial biofilm with selected phenolic compounds was grown in a microtiter plate at 37 °C for 24 h under static conditions. The WGA assay in the microtiter plate was performed as given in the protocol [41]. Fluorescence (λEx 495 nm and λEm 520 nm) was measured using a Varioskan Flash (Thermo Fisher Scientific, Waltham, MA, USA).

### 4.6. EPS Isolation

Overnight bacterial cultures of the tested strains were diluted into 10 mL TSB supplemented with 1% glucose at a 1:1000 ratio. Biofilms were grown overnight in 15 mL centrifuge tubes at 37 °C for 24 h under static conditions. After incubation, biofilms were pelleted by centrifugation at room temperature at 6000 rpm (Hettich^®^ Universal 320/320R—rotor 1620A) for 10 min. Next, each pellet was resuspended with 100 μL 1.5 M NaCl. The suspension was centrifuged again at room temperature, at 5000 rpm (Hettich^®^ Universal 320/320R—rotor 1620A) for 10 min, and the supernatant was used as the EPS fraction for further analysis.

### 4.7. Bradford Protein Assay

To determine the protein concentration in EPS fractions, we used the Bradford protein assay in microtiter 96-well plates. Aliquots of 20 μL EPS were poured into the wells and mixed with 180 μL Bradford Protein Assay Dye Reagent (Bio-Rad). The mixture was incubated for 5 min, and the Abs (595 nm) was measured by a Varioskan Flash (Thermo Fisher Scientific, Waltham, MA, USA).

### 4.8. Calorimetric DuBois Method

To determine the sugar content in the EPS fractions, we performed the DuBois calorimetric method [42] in microtiter 96-well plates. Briefly, 20 μL sample was mixed with 20 μL 5% phenol, 100 μL H_2_SO_4_ and incubated at room temperature for 10 min. Glucose was used as a standard, and Abs (492 nm) was measured by a microtiter plate reader (Varioskan Flash, Thermo Fisher Scientific, Waltham, MA, USA).

### 4.9. Confocal Laser Scanning Microscopy (CLSM)

To test cell viability, each biofilm culture was stained with DioC6 (3,3′-dihexyloxacarbocyanine Iodide, Merck, cat. no. 318426) at a final concentration of 0.02 μg/mL in order to image live cells and with propidium iodide (PI, Merck, cat. no. 25535-16-4) at a final concentration of 3 μg/mL to imagine dead cells. To image EPS components, WGA (Wheat Germ Agglutinin, Alexa Fluor™ 488 Conjugate, Thermo Fisher Scientific, cat.no. W11261) at a final concentration of 5 μg/mL was used to stain PIA/PNAG, and DAPI (4′,6-diamidino-2-phenylindole, dihydrochloride, Thermo Fisher Scientific, cat. no. D1306) and PI were used to stain eDNA.

For bacterial biofilm growth and analysis, Welco wells B.V. (Netherlands) Glass bottom Petri dishes were used. Treated or untreated biofilms were allowed to grow for 24 h, washed with PBS, and stained with appropriate dye. Microscopy was performed on a FluoroView FW1200 (Olympus LifeSciences), and images were adjusted and correlated by FluoroView FW1200 Software. Oil objective with 60x zoom was used for imaging.

### 4.10. Phenol-Chloroform RNA Extraction

Biofilms in a polystyrene Petri dish were grown and washed with PBS (pH 7.2) to remove planktonic cells. Nuclease-free water (1 mL) was added to the dish, and the biofilm was scraped off. Cells were transferred to a microcentrifuge tube and pelleted by centrifugation at room temperature at 13 400 rpm (MiniSpin^®^Eppendorf—rotor F45-12-11) for 5 min. Each pellet was resuspended with 10 μL RNase free water, after which 100 μL of a phenol:chloroform mixture at a 1:1 ratio was added. The mixture was incubated at 70 °C for 30 min with shaking, followed by centrifugation. The water fraction was then precipitated with isopropanol, and the pellet was washed with 70% ethanol two times, air-dried, and resuspended in nuclease-free water. The concentration of RNA was measured by NanoDrop (Thermo Fisher Scientific, Waltham, MA, USA).

### 4.11. Real-Time qPCR

To determine relative mRNA expression for each gene was performed in triplicates. A One-Step PCR kit (Qiagen), following a standard manufacturer’s protocol, was used. For all genes, primer annealing temperature was set to 60 °C, and for amplification, Roche LightCycler^®^ 480 96-well half-skirted plates were used. Real-time analysis was performed on the LightCycler^®^ 480 System by Roche. The housekeeping gene for 16S rRNA and template from nontreated samples were used as a control (Figure 10 and Figure 11). Primers are listed in Supplementary Materials (Table S1).

### 4.12. Statistical Analysis

All the measurements were performed in biological triplicate. Values as mean ± SD were obtained from Microsoft Excel, and statistical significance was evaluated by one-way ANOVA (*p* < 0.05).

## 5. Conclusions

The presented study demonstrates the potential usage of phenolic compounds from plant sources as natural antimicrobial agents against *S. epidermidis* biofilms. Vanillin and syringic acid were selected as phenolic compounds with no influence on the viability of bacterial cells and were tested on three representative strains with different genotypes. The effect of the two phenolic compounds on biofilm formation and EPS formation was determined and showed dose-dependent biofilm inhibition, resulting in a 70% decrease in biofilm mass at the highest concentration of vanillin or syringic acid. A similar inhibition effect was observed on EPS components, leading to the reduction in PIA/PNAG up to 40% of proteins and of eDNA independent of strain genotype, which can have a major effect on biofilm eradication. Vanillin and syringic acid also showed a QS inhibition effect by the lowering of the relative mRNA expression of selected *agr* QS mechanism genes, which can positively affect the production of virulent factors and other properties such as motility. These results suggest that natural phenolic compounds have properties to combat biofilms of methicillin-resistant *S. epidermidis* bacterial strains and in combination with antibiotics to help lower the prevalence of nosocomial infections.

## Data Availability

Not applicable.

## Supplementary Materials

The following supporting information can be downloaded at: www.mdpi.com/article/10.3390/ijms23031816/s1.
