# Supplementary material for: Complex Analysis of Vanillin and Syringic Acid as Natural Antimicrobial Agents against Staphylococcus epidermidis Biofilms
Source: Int J Mol Sci. 2022 Feb 5;23(3):1816. doi: 10.3390/ijms23031816 (PMC8836540; doi:10.3390/ijms23031816)
Supplement: Supplementary file 1 [file ijms-23-01816-s001.zip › ijms-1575435-supplementary.pdf]

Table S1. Primers used in this work.

Primer name	Sequence	Length	Reference
rt_aap_F	TGAGGCCGTACCAACAGTG	103 bp	This work
rt_aap_R	ATGGGCAAACGTAGACAAGGT		
rt_icaA_F	TGATCCTACGCACATCGCTT	94 bp	This work
rt_icaA_R	CGAACCACGTGCTCTATGCT		
hld_F	GACTCATTCAAAAATTATTTTTTG	100 bp	This work
hld_R	GAAGTTATAATGGCAGCAGATAT		
agrA_F	GTTTGTGAAGATGACCAAAGACA	104 bp	This work
agrA_R	AGGATCATTTGTTGCTAAAGC		
agrD_F	CACTACAATCTTGGAATTTATTGG	89 bp	This work
agrD_R	TCTGGTACTTCTGGTTCGTCAA		
PSMβ1_F	AGCAGCCATCACTAACG	90 bp	This work
PSMβ1_R	CCCAAAAATCGATTCACCATATC		
PSMβ2_F	GATGCAGGAATCAACCAAGATTG	93 bp	This work
PSMβ2_R	GACCTAATAATTTAGAAATAACA CTAATACC		
aap_F	AAACGGTGGTATCTTACGTGAA	466 bp	This work
aap_R	CAATGTTGCACCATCTAAATCAGC		
icaAB_F	CAATGCCGCAGTTGTC	543 bp	This work
icaAB_R	GTTTAACGCGAGTGCGC		
